# Physiological response of lower esophageal sphincter pressure to abdominal pressure in obese individuals: a retrospective cross-sectional study

**DOI:** 10.1590/0102-672020260000044e1973

**Published:** 2026-08-31

**Authors:** Yolanda Oliveira PINTO, Fernando Augusto Mardiros HERBELLA, Diego ADÃO, Leonardo de Mello DEL GRANDE

**Affiliations:** 1 Universidade Federal de São Paulo, Department of Surgery – São Paulo (SP), Brazil.

**Keywords:** Obesity, Esophagogastric Junction, Manometry, Esophageal Sphincter, Lower, Obesidade, Junção Esofagogástrica, Manometria, Esfíncter Esofágico Inferior

## Abstract

**Background:**

The physiological response of the lower esophageal sphincter (LES) to abdominal pressure (AP) plays a key role in the esophagogastric junction (EGJ) integrity. However, in obesity, this interplay remains unclear, particularly in the presence of anatomical alterations.

**Aims:**

This study aims to evaluate the correlation between abdominal pressure and LES basal pressure in obese patients, and to analyze the influence of EGJ morphology on this interaction.

**Methods:**

This retrospective cross-sectional study included 47 obese patients (BMI>35 kg/m^2^) who underwent high-resolution esophageal manometry. Patients were divided into two groups based on EGJ morphology: normal (Type I) and abnormal (Types II/III, hiatal hernia) and were compared based on clinical and manometric variables. Correlation analyses between AP and LES pressure were performed for the entire sample and stratified by EGJ morphology.

**Results:**

No statistically significant correlation was found between AP and LES pressure in the overall sample (rho=0.05; p=0.737). In the subgroup analysis, there was a non-significant trend toward increased LES pressure in patients with normal EGJ morphology (r=0.26; p=0.150) and decreased LES pressure in those with hiatal hernia (r=-0.39; p=0.131). No significant differences in BMI, abdominal circumference, AP, or LES pressure were observed between groups.

**Conclusions:**

In obese individuals, abdominal pressure does not show correlation with LES pressure. However, EGJ morphology may influence sphincteric behavior, with a trend toward LES pressure increase in normal EGJ and reduction in altered morphologies. These findings highlight the multifactorial nature of esophagogastric dysfunction.

## ARTICLE HIGHLIGHTS

No overall correlation was found between abdominal pressure and lower esophageal sphincter (LES) basal pressure in the studied obese population.

Patients with abnormal EGJ anatomy (hiatal hernia) demonstrated a non-significant trend toward decreased LES pressure under abdominal force.

The structural integrity of the EGJ plays a key modulating role, highlighting the multifactorial nature of barrier dysfunction in obesity.

## Summary

**CENTRAL MESSAGE:**

Obesity is a condition characterized by the abnormal accumulation of body fat and is a well-established risk factor for gastroesophageal reflux disease (GERD). Although the pathophysiology of gastroesophageal reflux disease is multifactorial, lower esophageal sphincter (LES) dysfunction is present in most patients with GERD, with a high prevalence in obese patients. From different and complementary pathways, raised intra-abdominal pressure from obesity seems to lead to a mechanical disruption of the gastroesophageal junction (EGJ), predisposing the patient to GERD. The response of the lower esophageal sphincter to increased intra-abdominal pressure is, however, not well understood when analyzing obese patients.

**PERSPECTIVES:**

In obese individuals, abdominal pressure does not show a consistent correlation with LES basal pressure. However, EGJ morphology may influence sphincteric behavior, with a trend toward LES pressure increase in normal EGJ and reduction in altered morphologies. These findings highlight the multifactorial nature of esophagogastric barrier dysfunction in obesity. Future studies incorporating other variables such as transdiaphragmatic pressure gradients, impedance-pH monitoring, and longitudinal follow-up may provide deeper insights into the mechanisms at play.

## VISUAL ABSTRACT

**Figure:**
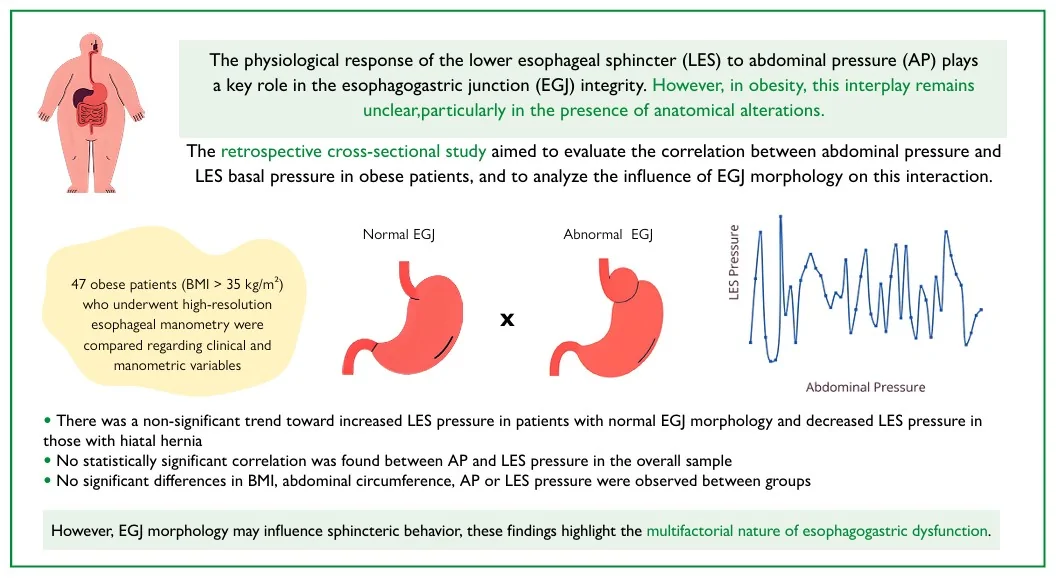

## RESUMO

**Racional:**

A resposta fisiológica do esfíncter esofágico inferior (EEI) à pressão abdominal (PA) desempenha um papel fundamental na integridade da junção esofagogástrica (JEG).

**Objetivos:**

Avaliar a correlação entre a PA e a pressão do EEI em pacientes obesos e a influência da morfologia da JEG nesta interação.

**Métodos:**

Realizou-se um estudo transversal retrospectivo incluindo 47 pacientes obesos (IMC>35 kg/m^2^) submetidos a manometria esofágica. Os pacientes foram divididos em dois grupos baseados na morfologia da JEG: normal (Tipo I) e anormal (Tipos II/III, hérnia do hiato) e comparados quanto às variáveis clínicas e manométricas. As análises de correlação entre a PA e a pressão do EEI foram realizadas para toda a amostra e estratificadas pela morfologia da JEG.

**Resultados:**

Não foi encontrada correlação entre a pressão abdominal e a do EEI na amostra geral (rho=0,05; p=0,737). Na análise de subgrupos, verificou-se uma tendência não significativa para o aumento da pressão do EEI nos doentes com JEG normal (r =0,26; p=0,150) e diminuição naqueles com JEG anormal (r=-0,39; p=0,131). Não foram observadas diferenças significativas no IMC, circunferência abdominal, PA ou pressão do EEI entre os grupos.

**Conclusões:**

Em indivíduos obesos, a PA não apresenta correlação com a pressão do EEI. Entretanto, a JEG pode influenciar o comportamento esfincteriano, com tendência para aumento da pressão na presença de JEG normal e redução da pressão em situações de morfologias alteradas. Estes achados destacam a natureza multifatorial da disfunção esofagogástrica.

## INTRODUCTION

 Obesity is a condition characterized by the abnormal accumulation of body fat and is a well-established risk factor for gastroesophageal reflux disease (GERD)^21^. Data from candidates for bariatric surgery revealed that reflux symptoms are present in over half of patients^18,21^, with a 5 kg/m^2^ increase in body mass index (BMI) associated with a 3-point rise in the DeMeester score17. Although the pathophysiology of gastroesophageal reflux disease is multifactorial, lower esophageal sphincter (LES) dysfunction is present in most patients with GERD, showing a high prevalence in obese patients^2 ,13,16^, although this association is not always statistically significant according to the literature^4,10,12,22^. 

 The pathophysiological response of LES pressure to increased abdominal pressure is still controversial. Increased abdominal pressure can lead either to an adaptive response in a competent sphincter or trigger valve dysfunction, mainly observed in those with concomitant hiatal hernia, compromising the anti-reflux barrier composed of the LES^7,19^. 

 From different and complementary pathways, raised intraabdominal pressure from obesity seems to lead to a mechanical disruption of the gastroesophageal junction (EGJ), predisposing the patient to GERD^14 ,15^. The behavior of the lower esophageal sphincter to increased intra-abdominal pressure is not, however, well understood when analyzing obese patients. 

 Thus, this study aims to evaluate the correlation between AP and LES pressure in the physiology of the EGJ in obese individuals. 

## METHODS

### Study design

 This cross-sectional study was conducted in the Department of Surgery at a single urban university hospital, where the data was obtained from electronic medical records covering the period from January 2016 to November 2024. The study also followed STROBE (Strengthening the Reporting of Observational Studies in Epidemiology) reporting recommendations^20^. 

### Ethics

 The Institutional Ethics Committee approved the study protocol (n^º^ 1037/2016). Written informed consent was obtained from all participants. No financial compensation was provided. The authors declare no conflicts of interest. All authors contributed significantly to the study and manuscript preparation. No professional writers were involved. This research did not receive any specific grant from funding agencies in the public, commercial, or not-for-profit sectors. No monetary compensation was provided to the individuals participating in the study. The authors declare no conflicts of interest. 

### Population

 Adults (>18 years) with grade II or III obesity (BMI>35 kg/m^2^), candidates for bariatric surgery, who had undergone high-resolution esophageal manometry (HRM), were included. 

### Clinical data and outcomes

 The following demographic and clinical data were recorded: sex, age, weight, height, waist circumference, and BMI. 

 All HRM tracings were reanalyzed by the same specialist using proprietary software (ManoScan and Manoview, Medtronic, Minneapolis, USA)^11^. The variables analyzed included abdominal pressure (AP), lower esophageal sphincter (LES) basal pressure, and morphology of the esophagogastric junction (EGJ). AP was measured 2 cm below the lower border of the LES and calculated as the mean pressure over a 30-second interval, encompassing all phases of respiration (mid-respiratory measurement). After the analysis of HRM, patients were divided into two groups based on EGJ morphology: group A (normal morphology, Type I) or group B (abnormal morphology, Types II and III). 

 The primary outcomes were the mean differences in LES basal pressure and AP (mm Hg). The secondary outcomes were BMI (kg/m^2^) and abdominal circumference (cm). 

### Statistical analysis

 Categorical variables were expressed using frequencies and percentages (%), and continuous variables as means and standard deviations (SD). Mean differences (MD) were reported considering a 95% confidence interval (CI) with robust standard errors. 

 Variables with normal distribution were analyzed using the independent samples Student’s t-test, while those without normal distribution were analyzed using the MannWhitney U test. 

 Correlation analyses between AP and LES basal pressure were performed for the entire sample and each group separately, using Pearson’s correlation coefficient for normally distributed variables, and Spearman’s rank correlation (rho) when the assumptions for Pearson’s test were not met. 

 For all analyses, p-values lower than 0.05 were considered statistically significant. 

 Statistical analyses were performed with the software Stata version 18. 

## RESULTS

 A total of 50 patients met the inclusion criteria. Three patients were excluded due to incomplete or unanalyzable HRM data, resulting in a final sample of 47 patients. Baseline data and manometric characteristics are presented in Table 1. 

**Table 1:** Clinical and manometric characteristics of the sample.

Variable		Mean (SD) or n (%)
Age (years)		43.7 (12.3)
BMI (kg/m^2^)		45 (7)
Sex		
	Male	9 (19)
	Female	38 (81)
Abdominal circumference (cm)		131 (13.6)
Abdominal pressure (mm Hg)		19.7 (6.1)
LES basal pressure (mm Hg)		17.4 (8.2)
EGJ Morphology		
	Normal (Type I)	31 (65.9)
	Hiatal hernia (Types II or III)	16 (34.1)

SD: standard deviation; BMI: body mass index; LES: lower esophageal sphincter; GEJ: gastroesophageal junction.

**Figure 1:**
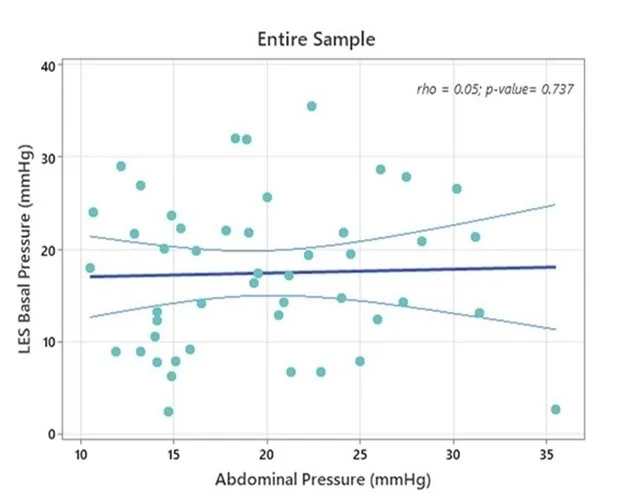
Correlation between lower esophageal sphincter (LES) basal pressure and abdominal pressure in the entire sample. The figure displays a fitted line representing the relationship between the variables, with a 95% confidence interval indicated by the two thinner lines. Spearman’s rank correlation (rho) coefficient and p-value are reported (p-value).

**Figure 2:**
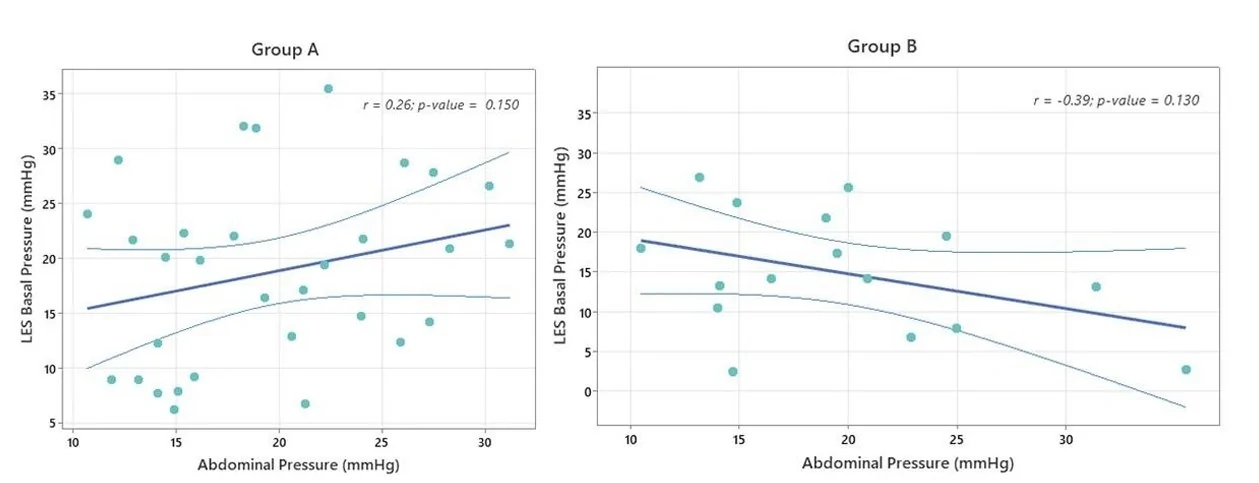
Correlation between lower esophageal sphincter (LES) basal pressure and abdominal pressure in Group A (normal hiatus morphology) and Group B (abnormal hiatus morphology). The figure displays a fitted line representing the relationship between the variables, with a 95% confidence interval indicated by the two thinner lines. The Pearson correlation coefficient (r) and corresponding p-value are reported (p-value).

 The primary outcome is reported in Table 2 and the findings graphically represented in Figures 1 and 2. For the total population, Spearman’s rank correlation test revealed no significant association (rho=0.05; p=0.737, p>0.05). In Group A, Pearson’s test indicated a weak positive correlation (r=0.26; p=0.150, p>0.05), while Group B showed a moderate negative correlation (r=-0.39; p=0.131, p>0.05). 

**Table 2:** Correlation between clinical and manometric variables by esophagogastric junction (EGJ) morphology.

Variable	Group A n=31 (65.9%) Mean (SD)	Group B n=16 (34.1%) Mean (SD)	Mean Difference (Group A–Group B) [95%CI]	p-value
BMI (kg/m^2^)	45.5 (7.2)	44 (6.7)	1.5 [-2.8–5.8]	0.686
Abdominal circumference (cm)	130.3 (12)	132.5 (16.4)	-2.2 [-11.8–7.4]	0.607
LES basal pressure (mm Hg)	18.7 (8.3)	14.9 (7.6)	3.8 [-1.1–8.7]	0.126
Abdominal pressure (mm Hg)	19.6 (5.9)	19.8 (6.8)	-0.2 [-1.1–8.7]	0.924

SD: standard deviation; BMI: body mass index; LES: lower esophageal sphincter; CI: confidence interval.

## DISCUSSION

 Contrary to the available data, no statistically significant correlation was observed between IAP and LES pressure in obese patients, even after stratification by EGJ morphology^9,13-15^. Changes in intragastric pressure, transient relaxations of the LES, and the gastroesophageal pressure gradient were previously explored in literature and may be involved in a complex interplay of multiple physiological variables which cannot be captured by pressure measurements alone^16^. 

 Although there were no statistically significant associations made, a trend toward increased LES pressure in individuals with normal EGJ morphology and decreased LES pressure in those with altered EGJ morphology was observed. The findings suggest that EGJ integrity may modulate the physiological relationship between abdominal and sphincter pressures^1,5,19^. In individuals with preserved EGJ morphology, the LES may retain its ability to respond to variations in intraabdominal pressure through a compensatory mechanism that contributes to the maintenance of the antireflux barrier^3,8^. Conversely, in patients with altered EGJ morphology, this adaptive response appears to be diminished, which may reflect a mechanical or neuromuscular impairment of the LES related to anatomical displacement, chronic distension, or changes in diaphragmatic support, which are commonly associated with hiatal hernias^6,7,19^. 

 These observations align with the hypothesis that the structural integrity of the EGJ is a key determinant of LES behavior under increased intra-abdominal pressure. Morphological disruption may not only compromise the pressure barrier itself but also interfere with reflexive regulatory mechanisms that ordinarily enhance LES tone in response to elevated abdominal forces^1 ,5,14^ . 

 Regarding the study design, the sample size may not have been able to detect more subtle associations. It is also essential to highlight that the study sample consisted of individuals who were candidates for bariatric surgery. The severe pathophysiological disturbances imposed on the body at extreme levels of obesity may be associated with impairment of standard autoregulatory mechanisms^9,11,13,17^. This potentially explains the absence of the expected correlation between increased intraabdominal pressure and LES, raising the possibility of a physiopathological threshold beyond which physiological responses are no longer activated appropriately or become dysregulated, independently of other variables such as EGJ morphology^1,5^. Furthermore, the cross-sectional nature of the analysis limits causal inference. 

 Individual variations in sphincter and mucosal integrity may modulate the role of central obesity and mechanical factors. Undoubtedly, the interplay between anatomical, functional, and possibly neurohormonal factors warrant further investigation into the multifactorial nature of esophagogastric barrier dysfunction in obesity. Future studies incorporating other variables such as transdiaphragmatic pressure gradients, impedance-pH monitoring, and longitudinal follow-up may offer deeper insights into the mechanisms at play. 

## CONCLUSIONS

 In obese individuals, abdominal pressure does not exhibit a consistent correlation with LES basal pressure. However, EGJ morphology may influence sphincteric behavior, with a trend toward LES pressure increase in normal EGJ and reduction in altered morphologies. These findings highlight the multifactorial nature of esophagogastric barrier dysfunction in obesity. 

## Data Availability

The datasets generated and/or analyzed during the current study are available from the corresponding author upon reasonable request.
